# A synthetic platform for developing recombinant adeno‐associated virus type 8 producer cell lines

**DOI:** 10.1002/btpr.70009

**Published:** 2025-02-19

**Authors:** Yu‐Chieh Lin, Han‐Jung Kuo, Min Lu, Thomas Mahl, George Aslanidi, Wei‐Shou Hu

**Affiliations:** ^1^ Department of Chemical Engineering and Materials Science University of Minnesota Minneapolis Minnesota USA; ^2^ The Hormel Institute Austin Minnesota USA

**Keywords:** adeno‐associated virus, biomanufacturing, gene therapy, HEK293, synthetic biology

## Abstract

Recombinant adeno‐associated virus (rAAV) is one of the most widely used viral vectors for gene therapy. It is used in very high doses for the treatment of many diseases, making large‐scale production for clinical applications challenging. We have established a synthetic biology‐based platform to construct stable production cell lines, which can be induced to produce rAAV2. In this study, we extended our cell line construction pipelines for rAAV2 to rAAV8, a serotype whose tropism makes it attractive for gene delivery in multiple tissues. The Genome Module, encoding the rAAV2 genome, and Replication Modules, containing Rep68, DBP and E4orf6 coding sequences, originally used for rAAV2 were retained, but the Packaging Module was modified to replace the AAV2 intron‐less *cap* gene (VP123) with that of AAV8. These three genetic modules were integrated into HEK293 genome to generate four rAAV8 producer cell lines VH1‐4, which all produced rAAV8 upon induction. Their productivity was similar to the initial rAAV2 producer cell lines GX2/6 constructed using the same pipeline, but was much lower than conventional triple plasmid transfection. We identified Cap protein production and capsid formation as a potential limiting factor, just as we observed in GX2/6. By integrating more copies of AAV8 VP123 into VH3 clone, the encapsidated rAAV8 titer increased 20‐fold to a level comparable to triple transfection. By tuning induction conditions to modulate capsid production, the full particle content could be elevated. This study demonstrated that our rAAV producer cell line development platform is robust and applicable to different AAV serotypes.

## 1 INTRODUCTION

Adeno‐associated virus (AAV) is a small and simple virus with a 4.7 kb single‐stranded DNA genome consisting of only two genes, *rep* and *cap*, flanked by two inverted terminal repeats (ITRs) and encased in a capsid. It cannot replicate alone by infecting a host cell, but requires coinfection with another virus, such as adenovirus, which provides helper functions, to replicate. Through alternative splicing and the use of alternative translation initiation, these two genes generate multiple transcripts and proteins. The large Rep proteins Rep78/68 and the small Rep proteins Rep52/40, which are essential for AAV genome replication and packaging, respectively,^1^, ^2^ are encoded by the *rep* gene. VP1, VP2, and VP3, encoded by the *cap* gene, form the capsid at a 1:1:10 ratio.^3^, ^4^ Assembly‐activating protein (AAP) and membrane‐associated accessory protein (MAAP), indispensable in AAV capsid assembly and secretion respectively, are also encoded by the *cap* gene.^5^, ^6^, ^7^

By replacing the *rep* and *cap* genes with a therapeutic gene of interest (GOI), the resulting recombinant AAV‐derived vector (rAAV) can be used safely and effectively to deliver target genes into a variety of human tissues with long‐term gene expression. The safety of AAV is attributed to its requirement for a helper virus for replication, while a number of AAV serotypes provide a range of tissue tropisms.^8^ In recent years, rAAV has become one of the most widely used in vivo system for gene therapy. Many rAAV‐based gene therapies have received the U.S. Food and Drug Administration (FDA) approval for clinical use for the treatment of retinal dystrophy, spinal muscular atrophy, hemophilia B, and Duchenne muscular dystrophy, underscoring the growing importance of rAAV in the field.^9^, ^10^

Conventionally, rAAV is produced by transient transfection of HEK293 cells with three plasmids. One plasmid encodes the rAAV genome; the second one contains the AAV *rep* and *cap* genes; the third carries the adenoviral helper genes *E2A*, *E4*, and *VA RNA*.^11^ Additionally, integrated into the genome of HEK293 are the adenoviral *E1A* and *E1B* helper genes. This method is commonly used and simple to implement for rAAV production. However, it requires additional production steps to produce multiple plasmids.^12^ Furthermore, a very large dose of rAAV, in the order of 10^13^–10^14^ vector genomes (VGs) per kg of a patient's weight, is used for in vivo systemic administration through intravenous injection.^13^, ^14^ Such a high dose requirement necessitates production in large scale, which poses challenges especially in cases where the patient population is large.

Others have developed adenovirus, baculovirus or herpes simplex virus (HSV) based vector system to produce rAAV.^15^ These viral vector systems use a packaging vector of the virus and two recombinant viral vectors, one carrying the rAAV genome and the other encoding the Rep and Cap proteins. The packaging vector is used to produce virus containing either the rAAV genome or the *rep*/*cap* gene. Next, the two viruses are used to coinfect the host cells to produce rAAV. In a more advanced baculovirus system, Rep and Cap protein stably incorporated into SF9 production cells and a single baculovirus was used with an expression cassette containing ITRs and GOI.^16^ Another approach created a stable HeLa cell line with AAV *rep* and *cap* genes integrated into the cell genome. This cell line produced rAAV upon coinfection with adenovirus.^17^ Although these systems are scalable, they require the production of viruses or helper virus for rAAV production. Furthermore, additional downstream process is necessary to remove the contaminating viruses as well as their nucleic acids and proteins.^15^

We have taken a synthetic biology approach to generate stable cell lines, which can be induced to produce rAAV2 without resorting to plasmid transfection and helper virus infection. The producer cell lines were constructed by integrating into HEK293 cell genome three genetic modules: a genome module (GM) encoding a green fluorescent protein (GFP) reporter gene flanked by AAV2 ITRs, a replication module (RM) containing inducible TetOn promoter‐driven Rep68 coding sequence (CDS) and adenoviral helper E4orf6 and DBP CDS, and a packaging module (PM) consisting of inducible CumateSwitch promoter‐driven AAV2 intron‐less *cap* gene and the Rep52 CDS.^18^, ^19^ An important design concept was the ability to modulate the kinetics of expression of key components in the system to optimize the productivity and quality of the rAAV produced. Additionally, GOI in the GM was placed under the control of an inducible Lac promoter to reduce the wasteful expression of GFP upon genome amplification during rAAV2 production.^19^ Through targeted quantitative proteomic analysis, we identified VP protein expression and capsid formation limiting the rAAV2 productivity in our cell lines, and more copies of PM were introduced to boost their capsid productivity.^19^, ^20^ Through design‐build‐characterization cycle, we have taken the approach from proof of concept^18^ to become a platform method for rAAV producer cell line construction and have shown that the cell lines had productivity on a par with conventional transient transfection‐based method.^20^

In the study reported here, we applied the platform method we developed for rAAV2 production to another serotype AAV8. In the PM, the inducible CumateSwitch promoter‐driven AAV2 intron‐less *cap* gene was replaced by that of AAV8 while retaining the AAV2 Rep52 CDS. AAV8 has tropism for liver, skeletal muscle, cardiac tissue, pancreas, and specialized cells in the brain and retina in mice.^21^ The therapeutic potential of rAAV8 vectors have been demonstrated in many animal studies.^21^ A recent clinical trial using rAAV8 vectors demonstrated the potential of gene therapy of factor IX gene in human liver.^22^ In contrast to AAV2, which remains largely intracellular after assembly and packaging, a significant fraction, up to 80%, of AAV8 particles are released into cell culture medium during production.^23^, ^24^, ^25^ Furthermore, a sevenfold higher of rAAV8 particles in the cell culture medium compared with the cell lysate was also described by Okada et al.^25^ These studies suggests that it is possible to harvest rAAV8 from the cell culture medium during production. However, the mechanism of AAV egress/release is not well understood. AAV8 MAAP has been observed to promote secretion of produced vectors through interaction with extracellular vesicles^6^; knockout of MAAP in AAV2 and AAV5 resulted in decreased virus egress.^26^

We report here that the pipelines originally developed for rAAV2 producer cell lines were successfully implemented in the development of rAAV8 producer cell lines. The kinetic behavior of the rAAV8 production by the newly constructed producer cell line was remarkably similar to that of rAAV2 producer cell lines. This work demonstrates that our cell line development platform can be used for the production of multiple AAV serotypes with consistent results.

## 2 MATERIALS AND METHODS

### 2.1 Vector construction

**FIGURE 1:**
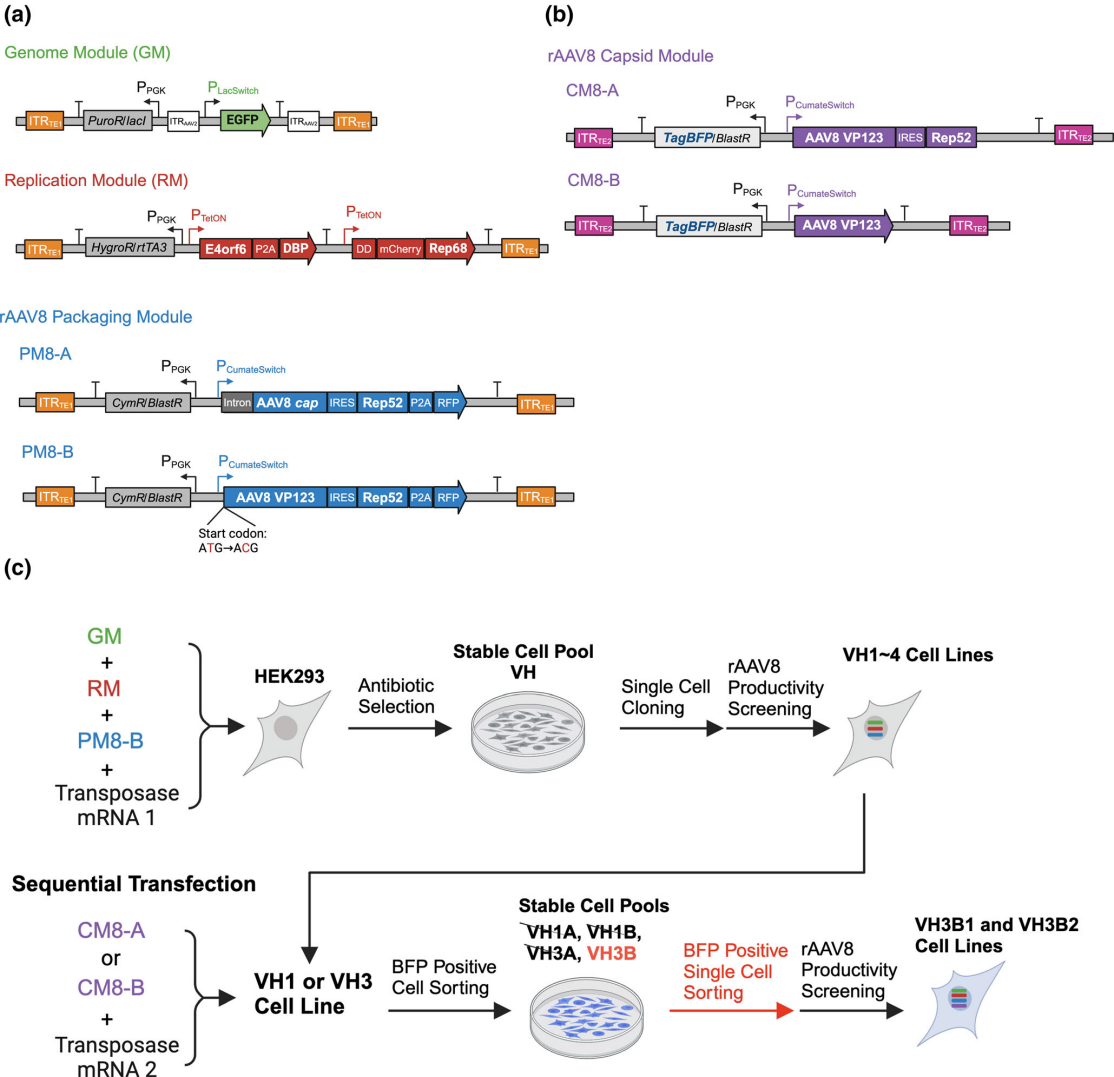
Schematic diagram of genetic modules used for (a) rAAV8 producer cell line construction and (b) enhancing VP protein and capsid productivity, and (c) rAAV8 production cell line development. (a) Genome module (GM) contains a cargo gene *GFP* coding sequence (CDS) under the control of inducible LacSwitch promoter flanked by AAV2 ITRs with a lacI repressor gene linked to a puromycin resistance gene driven by a phosphoglycerate kinase promoter (P_PGK_). Replication module (RM) contains an inducible TetOn promoter‐driven adenoviral helper E4orf6 and a DNA binding protein (DBP) CDS, and a destabilization domain (DD) and mCherry‐tagged AAV2 Rep68 CDS with a reverse Tet transactivator‐encoding gene *rtTA3* linked to a hygromycin resistance gene driven by a P_PGK_. rAAV8 Packaging Modules contain an inducible CumateSwitch promoter‐driven native AAV8 *cap* gene (PM8‐A) or intron‐less AAV8 *cap* gene (VP123) with an inefficient ACG start codon for VP1 (PM8‐B) linked to a Rep52 CDS via an IRES. In addition, the Rep52 CDS is linked through P2A to a small ultra‐red fluorescent protein (smURFP) CDS. Both PM8‐A and PM8‐B also contain a Cym repressor‐encoding gene *CymR* linked to a blasticidin resistance gene driven by a P_PGK_. Each genetic module was cloned into a transposon vector and flanked by the transposable element ITR 1 (ITR_TE1_), which was recognized by Transposase 1. (b) rAAV8 Capsid Modules CM8‐A and CM8‐B for sequential transfection of VP123 and Rep52 linked by IRES (CM8‐A) or VP123 alone (CM8‐B). Both contain a TagBFP CDS encoding a blue fluorescent protein for FACS. Each of these two modules was cloned into a transposon vector and flanked by the transposable element ITR 2 (ITR_TE2_), which was recognized by Transposase 2. (c) To construct rAAV8 producer cell lines, GM, RM, PM8‐B, and Transposase mRNA 1 were transfected into HEK293 cells and the three transposon vectors were integrated into the host cell genome. Following antibiotic selection, the VH cell pool was obtained. Single‐cell cloning and rAAV8 productivity screening were performed and four clonal cell lines, VH1 to 4, were isolated. VH1 and VH3 cells were chosen for sequential transfection with CM8‐A or CM8‐B. After rAAV8 productivity assessment on four resulting cell pools, the VH3B pool was chosen for further BFP positive single cell sorting. The rAAV8 productivity of the single‐cell clones was evaluated with RM4 assay cells. VH3B1 and VH3B2 cell lines were established. These figures were created with BioRender.com.

The GM and RM we previously used for rAAV2 producer cells were constructed as described (Figure 1a).^18^, ^19^ For the construction of rAAV8 packaging modules, native AAV8 *cap* CDS and intron‐less AAV8 *cap* CDS with an inefficient ACG start codon for VP1 were polymerase chain reaction (PCR)‐amplified from pAAV2/8 plasmid (Addgene #112864) and were joined to AAV2 Rep52 CDS via internal ribosome entry site (IRES) from encephalomyocarditis virus (ECMV) and placed under the control of the CumateSwitch promoter (System Biosciences, Palo Alto, CA, USA). The resulting DNA segments were then cloned into a Leap‐In transposon backbone containing a blasticidin resistance gene and the Cym Repressor, resulting in PM8‐A and PM8‐B, respectively (Figure 1a). PM8‐B was further modified to construct Capsid Modules CM8‐A and CM8‐B for the integration of more copies of the intron‐less AAV8 *cap* gene (Figure 1b). The Cym Repressor CDS in PM8‐B was replaced by a TagBFP CDS encoding a blue fluorescent protein to generate CM8‐A. Based on CM8‐A, the IRES and AAV2 Rep52 CDS were removed, yielding CM8‐B.

### 2.2 Generation of rAAV8 stable production cell lines

HEK293 cells obtained from Cell Biolabs Inc. (San Diego, CA, USA) were maintained in Dulbecco's Modified Eagle Medium (DMEM containing 4.5 g/L glucose; Gibco, USA) supplemented with 10% fetal bovine serum (FBS; Gibco, ThermoFisher, Waltham, MA, USA) and antibiotic/antimycotic (Gibco, ThermoFisher, Waltham, MA, USA) in T‐flask at 37°C in a 95% humidity and 5% CO_2_ air atmosphere. To develop synthetic rAAV8 producer cell lines, HEK293 cells were cotransfected with three transposon vectors, including GM, RM and PM8‐B, and Leap‐In Transposase® mRNA 1 (ATUM, Newark, CA, USA). Followed by 3 days of outgrowth, the cells were passaged and exposed to 2 μg/mL puromycin (Invivogen, San Diego, CA, USA), 200 μg/mL hygromycin B (MilliporeSigma, Burlington, MA, USA), and 10 μg/mL blasticidin (Invivogen, San Diego, CA, USA).^18^ After 2 weeks of antibiotic selection, rAAV8 production cell pool VH was obtained. Single‐cell cloning by limiting dilution and rAAV8 productivity screening using RM4 assay cell line^27^ were performed for the VH cell pool, yielding VH1‐4 clonal cell lines.

To increase the integrated copy number of the AAV8 *cap* gene, VH1 and VH3 cells were sequentially transfected with CM8‐A or CM8‐B and Leap‐In Transposase® mRNA 2 (ATUM, Newark, CA, USA). A BD FACSAria II Cell Sorter with a 100 μm nozzle was used for bulk sorting and single cell sorting of BFP‐positive cells.

### 2.3 rAAV production

For rAAV8 production using stable production cell lines, cells were seeded at 4 × 10^5^ cells per well in 6‐well plate (7.8 × 10^4^ cells/cm^2^). After 16 h, the culture media was replaced with induction media containing 10 μg/mL doxycycline and 90 μg/mL cumate (Sigma‐Aldrich, St. Louis, MO, USA). While for rAAV8 production using triple plasmid transfection, HEK293 cells were seeded at 7.5 × 10^5^ cells per well in 6‐well plate (1.56 × 10^5^ cells/cm^2^) and grown for 16 h, and followed by transfection of 2.5 μg of pAAV‐CAG‐EGFP (Addgene #37825), pAAV2/8 (Addgene #112864) and adenoviral helper gene‐carried pHelper (Cell Biolabs, USA) (Molar ratio = 1:1:1) using Transporter 5 transfection reagent (Polysciences, Warrington, PA, USA) (Figure S1a). For both production methods, cells and culture supernatant were harvested at various time points for isolation of rAAV8. The harvested cells were also used for isolation of intracellular DNA, RNA, and proteins.

### 2.4 rAAV preparation and titration

The AAV sample preparation for titration of encapsidated VG by quantitative polymerase chain reaction (qPCR) and capsids by enzyme‐linked immunosorbent assay (ELISA) were described previously.^18^ The primer pairs targeting cargo gene *GFP* were used for AAV8 VG titer quantification using qPCR.^18^ The pAAV‐CAG‐EGFP plasmid and AAV8 reference materials (RS‐AAV8‐FL, Charles River Laboratories, Hollister, CA, USA) were included as a positive control and used as a reference standard to determine the VG titer. The capsid titer was titrated using the AAV8 Titration ELISA kit (PRAAV8, PROGEN, Wayne, PA, USA). The total VG and total capsid titer included those harvested from cell lysate and from culture supernatant. The full particle content was determined by dividing the VG by the capsid titer.^19^, ^28^ Intracellular DNA was extracted using Quick‐DNA/RNA kits (Zymo Research, Orange, CA, USA) and subsequently used for the titration of intracellular total AAV genome (TG) copies by qPCR. Copies of TG were calculated with reference to the copies of *GPR15* in the host cell genome. Herein, VG represented the number of copies of the encapsidated rAAV genome, while TG represented the number of copies of both the unpackaged (free) and encapsidated rAAV genomes.

### 2.5 Targeted quantitative proteomics analysis

Protein sample preparation, parameters for acquisition of parallel reaction monitoring (PRM)‐based targeted mass spectrometry data, and raw data processing using Skyline were described in detail previously.^18^, ^19^, ^29^ The ratio of light to heavy peptides was calculated from the peak areas, and the ratios were then used to determine endogenous protein concentrations. New heavy isotope‐labeled peptides for AAV8 VP1, VP1/2/3 (sum of VP proteins), AAP, and MAAP quantification were designed and synthesized (Biosynth, Gardner, MA, USA). The sequence of peptide for AAV8 VP1/2/3 quantification is common to VP1, VP2, and VP3 of AAV8, while AAV8 AAP and MAAP were quantified using a peptide unique to each. As an internal control, human beta‐actin proteins encoded by *ACTB* (UniProt accession: P60709) were also quantified. A complete list of all the peptide standards used in this study was provided in Table S1. Viral protein copies per cell were calculated based on the assumption that the total protein of a HEK293 cell is 360 pg. The expression level of the viral protein was reported as the number of copies or molecules.

## 3 RESULTS

### 3.1 Higher rAAV8 productivity with packaging module containing intron‐less AAV8 cap gene

We extended our pipeline of constructing a synthetic stable rAAV producing cell line to serotype 8. The framework we adopted kept the AAV2 ITRs and *rep* gene, changed only the *cap* gene to serotype 8.^30^ Notably, AAV2 Rep52 was used to package the AAV2 VG into an AAV8 capsid to produce a pseudotyped rAAV. Hence, the GM and RM that we constructed for rAAV2 production (Figure 1a) were retained, and the packaging module was changed to be for rAAV8. Two new packaging modules for AAV8 were constructed. The first one had a native AAV8 *cap* gene containing the intron region, and the other contains intron‐less AAV8 *cap* gene (VP123) with an inefficient ACG start codon for VP1. Each was linked to AAV2 Rep52 by IRES and placed at the downstream of CumateSwitch promoter, resulting in rAAV8 packaging modules PM8‐A and PM8‐B, respectively (Figure 1a).

**FIGURE 2:**
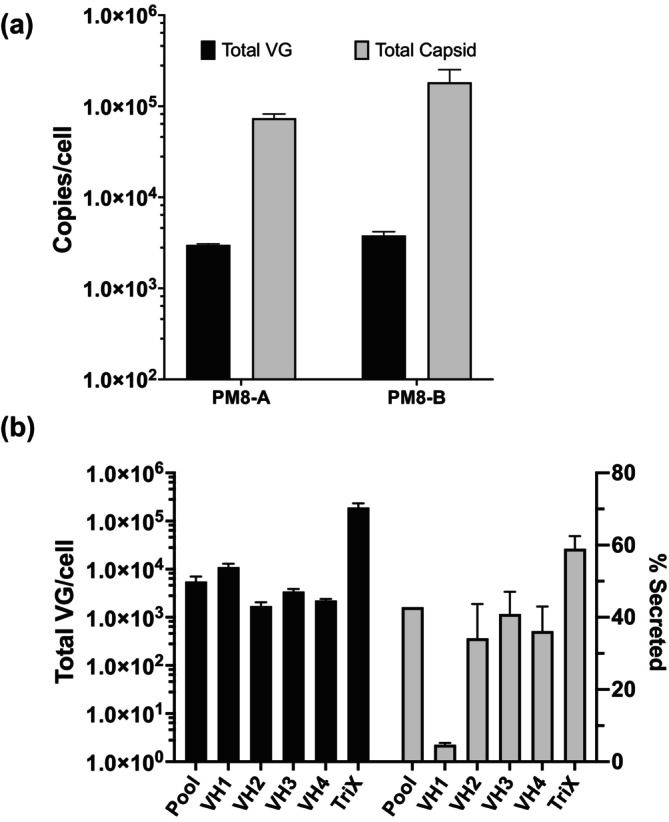
Selection of packaging modules for rAAV8 and titer of the resulting rAAV8 producer cell lines. (a) Total VG and capsid of produced by HEK293 cells transiently transfected with GM, RM and PM8‐A or PM8‐B and induced with 10 μg/mL doxycycline and 90 μg/mL cumate (10D90C) for 72 h. The total VG and capsid titer included rAAV8 from both cell lysate and culture supernatant. (b) The total VG titer (left *y*‐axis) and percentage of total VG secreted to culture supernatant (right *y*‐axis) of VH cell pool, VH1‐4 clonal cell lines induced with 10D90C and TriX. All the samples were harvested at 72 h post induction or transfection. The data are presented as the means ± SDs (*n* = 3).

HEK293 cells were transiently transfected with GM, RM and PM8‐A or PM8‐B under induction by doxcycline (D) at 10 μg/mL and cumate (C) at 90 μg/mL. After transfection and induction for 72 h, both cells and culture supernatant were harvested for the titration of AAV8 capsids and encapsidated VG. As shown in Figure 2a, with either PM8‐A or PM8‐B, assembled AAV8 capsids were present in both culture supernatant and intracellularly. AAV8 capsid and VG titers were both higher with PM8‐B construct (Figure 2a). Therefore, PM8‐B was chosen for the construction of rAAV8 stable producer cell lines.

### 3.2 Construction of rAAV8 stable production cell line

The GM, RM, and PM8‐B were transfected into HEK293 cells and integrated into host cell genome to generate VH cell pool (Figure 1c). This was followed by single cell cloning and rAAV8 productivity screening to obtain clonal stable VH producer cell lines 1–4 (Figure 1c). The total VG titers after induction for 72 h, including those harvested from cell lysate and from culture supernatant, of the VH pool and four cell lines VH1‐4 are shown in Figure 2b. Their titer reached 10^3^–10^4^ VG/cell levels; however, it was still approximately an order of magnitude lower than that of the traditional rAAV8 triple plasmid transient transfection of HEK293 cells (TriX) (Figures S1a and 2b). In addition, the proportion of VG secreted into the media was also lower than the TriX (Figure 2b).

### 3.3 VP protein level and capsid productivity

**FIGURE 3:**
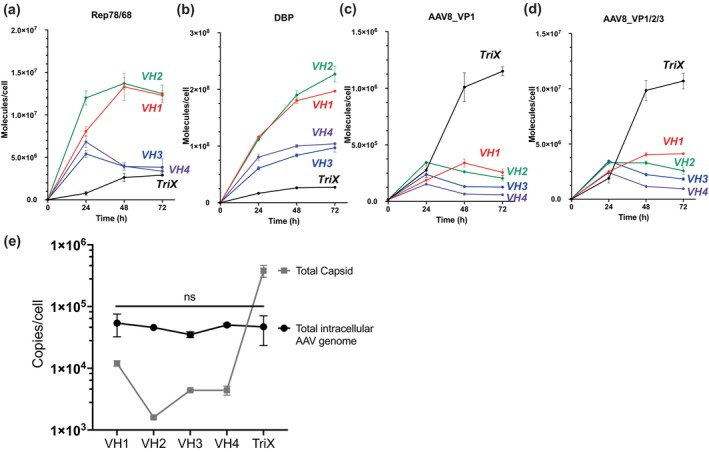
Time dynamics of viral proteins, capsid, and total intracellular AAV genome in the production of rAAV8. VH1‐4 clonal cell lines were induced with 10D90C. The producer cell lines and TriX are as labeled on the graphs. Cells were harvested at various time points for absolute protein quantification of (a) Rep78/68, (b) DBP, (c) AAV8_VP1, (d) AAV8_VP1/2/3 (sum of VP proteins) using targeted quantitative proteomics. The total capsid and total intracellular AAV genome are in (e). The data are presented as the mean ± SDs (*n* = 3). ns: no significant different (*p* > 0.05, Student's *t*‐test).

Absolute quantification (AQUA) of viral proteins, including Rep78/68, DBP, AAV8 VP1/2/3 (sum of VP proteins), and AAV8 VP1, was done by targeted quantitative proteomics. Notably, AAV8 VP1/2/3 was quantified using a peptide common to VP1, VP2, and VP3, while AAV8 VP1 was quantified using a peptide unique to it (Table S1). As shown in Figure 3a–d, the protein level of Rep78/68 and DBP in all four VH cell lines were comparable and even higher than that of TriX, whereas the protein level of VP1 or VP1/2/3 was at least an order of magnitude lower than that of TriX.

The higher Rep78/68 and DBP protein expression in VH cell lines did not result in higher TG production. After induction or transfection for 72 h, the TG titer of VH1‐4 and TriX reached 10^4^–10^5^ copies per cell (Figure 3e). The lower VP protein level in VH1‐4 cell lines was reflected in their capsid titer. The capsid titer of VH1‐4 was 50–100‐fold lower than that of TriX (Figure 3e). In terms of capsid assembly rate, approximately 75% of the VP1/2/3 proteins were assembled as AAV8 capsids in TriX, whereas only 20%–30% of VP proteins were assembled as capsid in VH cell lines. Taken together, the results suggested that AAV8 VP proteins and capsid production may be insufficient in VH cell lines.

### 3.4 Boosting VP protein and capsid production enhance rAAV8 productivity of PM8 clones

**FIGURE 4:**
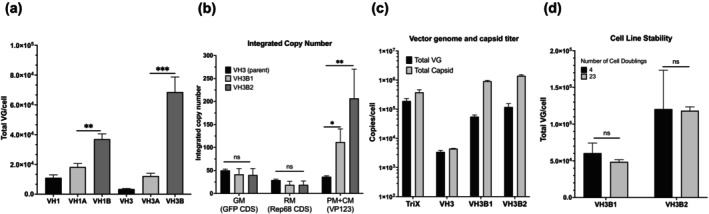
Comparable rAAV8 productivity upon boosting VP production. (a) The total VG titer of VH1, VH3 and their derived CM8‐A and CM8‐B‐integrated cell pools VH1A, VH1B, VH3A, and VH3B. (b) The integrated copy number of GM, RM and sum of PM and CM in parent VH3 and two derived clones, VH3B1 and VH3B2. The integrated copy number of each gene module was measured using qPCR by targeting GFP CDS, Rep68 CDS, and AAV8 VP123, respectively, and in reference to host cell *GRP15* gene. (c) The total VG and capsid titer of TriX, VH3, VH3B1, and VH3B2. (d) The total VG titer of VH3B1 and VH3B2 before and after 19 cell doublings. *p < 0.05, **p < 0.01, ns: no significant different (*p* > 0.05, Student's *t*‐test).

Based on the results shown in Figure 3, enhancing VP protein synthesis and increasing capsid production could possibly enhance rAAV8 production in the VH clonal cell lines. rAAV8 Capsid Modules CM8‐A and CM8‐B were constructed for integrating more copies of AAV8 VP123 to VH clones (Figure 1b,c). CM8‐A contained a CumateSwitch‐driven AAV8 VP123 and AAV2 Rep52 CDS, while CM8‐B contained only CumateSwitch‐driven AAV8 VP123 CDS (Figure 1b). Both modules contained a TagBFP encoding blue fluorescent protein as a reporter for fluorescence‐activated cell sorting (FACS) (Figure 1b). CM8‐A or CM8‐B was integrated to VH1 and VH3 clones using transposase, and BFP positive cells were sorted into cell pools (Figure 1c). After induction for 72 h, the total VG titers of either CM8‐A or CM8‐B‐integrated VH1 and VH3 cell pools, including VH1A, VH1B, VH3A, and VH3B, were higher than their untransfected parental cells (Figure 4a). Moreover, the total VG titers of CM8‐B‐integrated VH1 and VH3 were significantly higher than those integrated with CM8‐A (**p < 0.01, ***p < 0.001) (Figure 4a). The results showed that increasing the integrated copies of AAV8 VP123 in both VH1 and VH3 boosted rAAV8 productivity markedly. In addition, increasing the copy number of integrated copies of VP123 alone, gave a better VG titer than coamplifying both VP123 and Rep52. The cell pool which had the highest total VG titer in Figure 4a, VH3B, was chosen for further investigation.

### 3.5 Comparable rAAV8 productivity as triple transfection upon boosting VP production

Two clones, VH3B1 and VH3B2, were isolated from VH3B cell pool. Both clones had more copies of VP123 integrated into host cell genome (including both PM8‐B and CM8‐B) than the parent VH3, while the copy number of GM and RM remained at similar levels as VH3 (Figure 4b). Both clones with boosted copies of VP123 produced more capsids than their parental clone VH3 and their capsid titers were comparable to that of TriX (Figure 4c). With the increased capsid titer, the total VG titer of clone VH3B2 reached 1.2 × 10^5^ copies per cell, which is about 20‐fold higher than that of its parental clone VH3 (Figure 4c).

In addition, both VH3B1 and VH3B2 clonal cell lines are stable in terms of the rAAV8 productivity, the total VG titer was sustained at same levels after about 20 cell doublings (Figure 4d). Twenty population doublings represent an approximately 10^6^ fold increase in cell number, an amount sufficient to expand from a 100 mL culture to a 10 m^3^ or larger bioreactor. The data suggest that the genetic modules integrated into the cellular genome were structurally stable and did not appear to be epigenetically silenced during culture. The AQUA data of the parent cell clone VH3 showed that without induction, the protein level of Rep68 which was growth inhibitory and could be a source of genetic instability was very low (Figure 3a). The absence of high levels of leaky expression of Rep68 probably contributed to the stability (Figure 3a). We noted that in industrial practice for recombinant protein production, stability is tested over an even longer period.^31^ Since the cell lines we generated were model cell lines with *GFP* as GOI, we did not perform such a long‐term stability test.

### 3.6 Altered induction dynamics of capsid production affected full particle content

**FIGURE 5:**
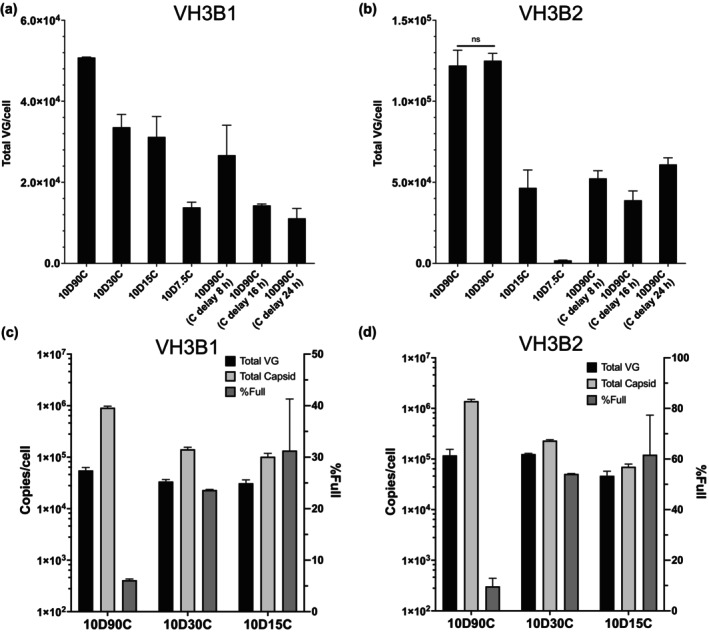
Altered induction dynamics of capsid production affected full particle content. For both VH3B1 and VH3B2 cell lines, the induction concentration of cumate was varied and its induction time was delayed by different periods, while both doxycycline (denoted as d) and cumate (denoted as c) induction were terminated at 72 h. The total VG titers of both cell lines with different induction conditions were shown in (a) and (b), respectively. The total VG titer, total capsid titer and full particle content (%full) of both cell lines induced with 10D90C, 10D30C, and 10D15C were shown in (c) and (d), respectively. ns: no significant different (*p* > 0.05, Student's *t*‐test).

As shown in Figure 4c, VH3B1 and VH3B2 cells had comparable encapsidated rAAV8 productivity as triple transfection. However, the full particle contents of rAAV8 produced by these two VH3B cell lines were only 5%–10% (Figure 5c,d). After induction, both VH3B1 and VH3B2 produced high levels of AAV genome, and even higher levels of capsid resulting in huge excess of capsids, which were left unencapsidated. An important feature of our modular design was the capability of independent manipulation of the induction of Rep68/DBP/E4orf6 and Cap/Rep52. We explored the effectiveness of reducing capsid formation by reducing or delaying Cap protein production to shift the stoichiometric unbalance and reduce the empty capsids. The induction concentration of cumate was varied or its induction initiation was delayed by various time period. With 10 μg/mL doxycycline (10D) induction, reducing the induction cumate concentration from 90 (90C) to 30, 15, and 7.5 μg/mL (30, 15, and 7.5C, respectively) and delaying cumate induction by 8, 16, and 24 h, while terminating induction both at 72 h, reduced the total VG titer in both VH3B1 and VH3B2 cells to different extents (Figure 5a,b). Notably, the total VG titer of VH3B2 cells did not significantly decrease under 10D30C induction, whereas decrease the cumate induction concentration to 7.5C and delaying cumate induction by more than 8 h would severely decrease the total VG titer of both clones (Figure 5a,b).

The kinetics of capsid and genome production with different cumate induction concentrations showed that reducing cumate concentration reduced capsid formation (Figure S2a–f). At 72 h, the capsid titer under 10D30C and 10D15C induction was approximately 10‐fold lower (Figure 5c,d). Interestingly, the TG with reduced cumate induction concentration was somewhat higher in both clones (Figure S2a–f). One may speculate that with a lower level of cumate induction and the reduced capsid formation, more resources may be available to generate more AAV genomes. This reduced total capsid titer but comparable or even higher TG levels resulted in increased full particle content in both VH3B1 and VH3B2 (Figure 5c,d). Notably, VH3B2 induced with 10D30C had over 50% full particles (Figure 5d).

### 3.7 Expression of viral proteins in VH3B1 and VH3B2 clonal cell lines

The rAAV8 productivity of VH3B1 and VH3B2 was comparable to that of conventional rAAV8 triple transfection. However, the two clonal cell lines had rather different rAAV8 secretion behavior. The triple transfection gave rise to approximately 50% of secreted encapsidated rAAV8 particles in the culture media. The parent clone VH3 had approximately 40% secreted, whereas only a very small fraction of rAAV8 was secreted in VH3B1 and VH3B2 (Figure S3).

The expression profile of AAV and adenoviral helper proteins was quantified in TriX, VH3 (parent), and VH3B1 and VH3B2 clonal cell lines using AQUA. The schematic diagram of the coding sequences of the proteins in the *cap* gene was shown in Figure S1c. Human beta‐actin proteins encoded by *ACTB* were also quantified and used as an internal control herein (Figure S4e–h).

**FIGURE 6:**
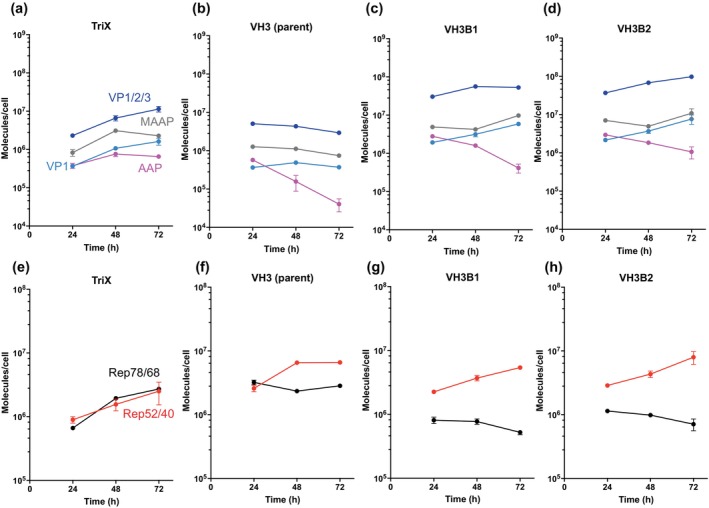
Expression of AAV proteins in TriX, VH3, VH3B1, and VH3B2 cell lines. (a, e): rAAV8 triple plasmid transient transfection of HEK293 cells (TriX); (b, f): VH3 parent cell line; (c, g): VH3B1 cell line; and (d, h): VH3B2 cell line. The data are presented as the means ± SDs (*n* = 3).

With additional copies of VP123 integrated in their genome, both VH3B1 and VH3B2 cell lines had increased level of VP1 and total VP proteins compared with their parent clone VH3. The level was even higher than in TriX (Figure 6a–d). In addition, Western blotting showed no clear difference in the relative abundance of VP1, VP2, and VP3 among the two clones, their parent clone VH3 and TriX (Figure S5).

The expression of AAP protein in VH3B1 and VH3B2 reached peak value early at 24 h at levels somewhat higher than in TrX and VH3, and declined subsequently (Figure 6a–d). AAP protein level did not suggest that AAP being a limiting factor for the lack of rAAV8 secretion in VH3B1 and VH3B2 (Figure 6a–d).

AAV8 MAAP has been reported to be a viral egress factor.^6^ Knockout of MAAP in wild‐type AAV8 markedly attenuated AAV release into the medium and increased intracellular retention.^6^ As shown in Figure 6a–d, MAAP was expressed at high levels of 10^6^–10^7^ molecules per cell, but still much lower than VP1/2/3. The expression was higher in VH3B1 and VH3B2 than in TriX and the parent VH3. MAAP promotes the interaction between AAV8 particles and extracellular vesicles and facilitates AAV8 secretion.^6^ Considering that approximately 60 copies of VP1/2/3 form one capsid particle, and the capsid titer was about 10^6^ copies per cell in VH3B1 and VH3B2, the copy ratio of MAAP to capsid is high, approximately 10:1, while the ratio in TriX was nearly 15:1.

In both VH3B1 and VH3B2, the Rep68 protein was not highly expressed as other viral proteins (Figure 6e–h). The adenoviral helper protein DBP was the most highly expressed of all viral proteins, while E4orf6 was expressed only at lower levels (Figure S4a–d), similar to what we previously observed in the synthetic cell lines GX6A and GX6B for rAAV2 production.^20^

## 4 DISCUSSION

In this study, we set out to use the latest cell line construction pipeline established for rAAV2 to construct stable cell lines for the production of an rAAV of serotype 8.^18^, ^19^, ^20^ A study aim was to test whether the pipeline for rAAV2 would also create rAAV8 cell lines with comparable productivity and other characteristics. We took our latest design of the three genetic modules for our GX cell lines^19^, ^20^ as the framework for the construction of rAAV8 synthetic cell lines, keeping the same Genome Module and Replication Module which used ITR and Rep68 CDS of rAAV2. Only in the Packaging Module, the intron‐less *cap* gene of AAV2 was replaced by the one from AAV8. Therefore, the produced rAAV was an AAV8 capsid encapsidating a genome flanked by AAV2 ITRs. Although this was our first use of the synthetic cell line construction pipeline for a pseudotyped rAAV, similar use of pseudotyped rAAV has been used to produce rAAV1‐6, ‐8, ‐9, and ‐rh.10 in transient transfection of 293T or HEK293 cells.^30^, ^32^ Note that in this approach, Rep52 of AAV2 would act on packaging VGs in capsids of another AAV serotype, in this case AAV8. AAV2 Rep has been shown to be capable of packaging VGs into AAV capsids of different serotype, although the packaging efficiency was lower for some capsids compared with AAV2.^33^ It is possible that the efficiency would be better if the Rep52 and VP proteins were of the serotype 8. We did not explore the use of AAV8 Rep52 CDS in our cell line construction since others have shown in transient transfection‐based rAAV production that such systems with mixed serotype gene components are functionally sufficient^32^, ^33^ and the productivity of our approach of using a mixed serotype viral component was high.

While developing the Packaging Module for constructing synthetic cell lines for rAAV2 production,^18^ we adopted the same intron‐less AAV2 *cap* gene construct VP123 with translational read‐through enabled by an inefficient ACG start codon for VP1 as reported by Urabe et al.^34^ Such a construct allowed the three VP proteins, VP1, VP2, and VP3, to be expressed from a single expression cassette and allowed the expression level of these three proteins to be close to the apparent protein ratio in the AAV capsids, 1:1:10.^34^ The adoption of VP123 construct significantly increased the titer of rAAV2 compared with that with the native AAV2 *cap* gene.^18^

The *cap* gene of AAV8 had 83% identity with that of AAV2.^32^ The intron splice site and translation initiation sites of AAV8 *cap* gene were all similar to that of AAV2. Therefore, we used the same approach of engineering *cap* gene developed for AAV2 for our AAV8 *cap* gene. In the case of AAV8, we found that the VP123 construct of AAV8 also enhanced capsid and encapsidated rAAV productivity compared with the native *cap* construct.

The pipeline for rAAV2 producer cell line construction was efficient in generating cell pools with a high proportion of high‐producing clones. With the rAAV productivity screening assay using the RM4 cell line,^27^ those good producers could be identified and isolated. While confident in the effectiveness of the pipeline to construct the rAAV2 producer cell lines, it was reassuring that the pipeline worked well when applied to construct the rAAV8 producer cell lines. Interestingly, the results of the two cell line construction studies were very similar, as summarized in Figure S6. The copy numbers of the three genetic modules integrated into the host cell genome were in a similar range between rAAV2‐producing GX2/GX6 and rAAV8‐producing VH3. All in the range of 20–50, with the Replication Module being somewhat lower than the other two. The GX2/6 and VH3 cell lines were also similar in their titers. Both groups of cells (GX and VH) yielded approximately 3 × 10^3^ VG/cell, 3 × 10^3^–10^4^ capsid/cell. In addition, both GX and VH clones appeared to have a common limiting factor for increasing productivity, insufficient VP proteins and capsids. To increase the titer of encapsidated rAAV particles, the integrated copies of VP123 were increased to boost capsid production, resulting in enhanced VG titers in both GX6A/B and VH3B1/2. However, the increased VG titer came at the expense of increased empty particle content in GX6A/B and VH3B1/2.

In the present study, our comparison of coamplification of Rep52 CDS and VP123 with amplification of VP123 alone suggested that increasing the expression of Rep52 protein was not beneficial for packaging. In addition, we noted that CM8‐B, without the IRES and Rep52 segment, was about 2 kilobase smaller than CM8‐A (Figure 1b). The size of plasmid DNA affects its transfection efficiency.^35^, ^36^ The small size of CM8‐B may have contributed to the higher integrated copy number of VP123 in VH3B1/2 than in GX6A/B (Figure S6).

Another similarity, perhaps less surprising, between the two parallel cell line developments was the tunability of VP protein production and capsid formation by adjusting cumate induction conditions. Reducing the excess capsid production in the final VP123‐amplified cell lines improved rAAV full particle content. It is plausible that increasing TG productivity can further enhance their encapsidated rAAV2/rAAV8 productivity and full particle content in both GX6A/B and VH3B1/2. However, increasing the level of Rep68, Rep78 and helper proteins in VH3B1/2 did not drastically increase the total VG titer (Figure S7). In our previous study, Shield1 was added to the culture of GX6A cells to stabilize and increase the level of Rep68 proteins during rAAV2 production, but no positive effect on rAAV2 VG titer was observed.^20^

In the transfection‐based production system, a significant proportion, reported up to 78.4%, of the rAAV8 produced was released into the culture supernatant after 5 days of transfection.^24^ MAAP plays a key role in the secretion of AAV8.^6^ The rAAV8 producer cell lines VH1‐4 secreted nearly 40% of the viral particles. Targeted proteomic data showed that VH3 produced MAAP at high levels of approximately 10^6^ molecules per cell (Figure 6b), suggesting that VH3 had all the necessary cellular and viral components to secrete rAAV8. It was therefore rather unexpected that VH3B1 and VH3B2, both of which had boosted VP protein expression and higher MAAP levels than VH3 (close to 10^7^ copies/cell) (Figure 6b–d), almost abolished rAAV8 release (Figure S3). The possible reasons for the lack of secretion in VH3B1/2 are many. Compared with VH3, VH3B1/2 had higher levels of capsids and MAAP, but the molar ratio of MAAP to capsids was actually nearly tenfold lower. It was possible that the facilitating role on secretion of MAAP is affected by the kinetic or stoichiometric interactions with capsids or some other cellular component. It is also plausible that MAAP, while necessary, still requires the participation of some unidentified factors for AAV8 secretion. A transient transfection experiment was conducted to probe viral components, which could enhance the secretion of rAAV8 particles (Figure S7). VH3B2 cells were transfected with pAAV2/8, pHelper, or CDS of individual AAV2 Rep or AAV8 Cap proteins. Transfection with both pAAV2/8 and pHelper plasmids together markedly increased VG secretion to ~60% levels, similar to the levels seen with triple transfection of HEK293 cells (Figure S7). This increase in secretion required both pAAV2/8 and pHelper. Either one alone did not boost the secretion of VG to beyond 10% (Figure S7). Transfection with individual CDS of AAV8 AAP, MAAP and of AAV2 Rep proteins (Rep78, Rep68, Rep52, and Rep40) did not improve secretion either (Figure S7). Therefore, VH3B2 likely requires some unidentified factor(s) that is either missing or out of balance in VH3B2 cells but is present in pAAV2/8 and pHelper. We noted that the two plasmids which carried segments of native *rep*, *cap* and adenoviral helper genes also included the intergenic regulatory regions. Our genetic modules retained only the viral protein CDS without any intergenic region. Some of the “missing” element(s) may reside in the intergenic region of native viral sequences of pAAV2/8 and pHelper.

rAAV of several serotypes, including AAV1, AAV6, AAV8, and AAV9, are capable of being released to the culture supernatant during production.^23^, ^24^ In some cases, these viral particles are secreted efficiently.^23^, ^24^, ^25^ In general, the amount of viral particles secreted and retained intracellularly were in the same order of magnitude. It is therefore customary that viral particles were harvested from both pelleted cells and culture supernatant. Dealing with a relatively dilute supernatant stream and a much more concentrated cell lysate product stream presents a challenge for downstream processing. An ideal rAAV production process is to either to secrete almost all or to retain all intracellularly.^37^ From this perspective, the VH3 cell line, which secretes approximately 40% of the viral particles, would likely require product recovery from both the culture supernatant and cell lysate. Therefore, retaining almost all rAAV8 particles intracellularly in VH3B1/2 cells might not be so undesirable.

## 5 CONCLUSIONS

In conclusion, the cell line construction pipeline developed for rAAV2 was applied to develop rAAV8 producer cell line, which yielded surprisingly similar results. The cell lines produced approximately 2–20 × 10^4^ VG/cell depending on the induction conditions, which could be tuned to generate viral particles with higher full particle content but slightly lower VG/cell. Both rAAV8 titer and the full particle content could potentially be improved by screening more candidate clones to increase the probability of obtaining a clone with high VG and full particle content. Adaptation of these cell lines to suspension culture, as we have shown in synthetic rAAV2 production cell lines, would make the production more amenable to large scale operations.^20^ Optimization of process conditions like medium formulation, induction profile and possibly adoption of high cell‐density perfusion culture as commonly practiced in therapeutic protein production,^38^, ^39^ will likely enhance productivity and product quality further.

## AUTHOR CONTRIBUTIONS

**Yu‐Chieh Lin:** Conceptualization; writing – review and editing; writing – original draft; project administration; data curation; methodology. **Han‐Jung Kuo:** Writing – original draft; writing – review and editing; project administration. **Min Lu:** Conceptualization; writing – review and editing; project administration. **Thomas Mahl:** Project administration; writing – review and editing. **George Aslanidi:** Conceptualization; writing – review and editing. **Wei‐Shou Hu:** Supervision; conceptualization; writing – original draft; writing – review and editing; funding acquisition.

## CONFLICT OF INTEREST STATEMENT

The authors declare no conflict of interest.

## Supporting information


**Data S1.** Supporting Information.

## Data Availability

The data that support the findings of this study are available on request from the corresponding author. The data are not publicly available due to privacy or ethical restrictions.
