# Supplementary material for: A synthetic platform for developing recombinant adeno‐associated virus type 8 producer cell lines
Source: Biotechnol Prog. 2025 Feb 19;41(3):e70009. doi: 10.1002/btpr.70009 (PMC12171320; doi:10.1002/btpr.70009)
Supplement: Supplementary file 1 — Data S1. Supporting Information. [file BTPR-41-e70009-s001.docx]

**Supporting Information**

**Development of recombinant adeno-associated virus type 8 stable producer cell lines**

Yu-Chieh Lin^1^, Han-Jung Kuo^1^, Min Lu^1^, Thomas Mahl^1^, George Aslanidi^2^, Wei-Shou Hu^1,^*

^1^Department of Chemical Engineering and Materials Science, University of Minnesota, 421 Washington Avenue S.E., Minneapolis, Minnesota 55455-0132

^2^The Hormel Institute, Austin, MN 55912, USA

*CORRESPONDING AUTHOR

Wei-Shou Hu

Address: 421 Washington Avenue SE, Minneapolis, MN 55455-0132 USA

Phone: (612) 626-7630

Fax: (612) 626-7246

Email: wshu@umn.edu

**Figure S1. Schematic diagram of (A) rAAV8 triple plasmid transient transfection of HEK293 cells, (B) CisReg43CMV-bGpA expression vector, and (C) the alternatively spliced transcripts and protein coding sequences of AAV *rep* and *cap* genes.** (A) For rAAV8 production using triple plasmid transient transfection, HEK293 cells were equally transfected with pAAV-CAG-EGFP containing cargo gene *GFP* flanked by AAV2 inverted terminal repeats (ITRs), pAAV2/8 plasmid containing AAV2 *rep* and AAV8 *cap* genes, and pHelper plasmid containing adenoviral helper genes *E2A*, *E4* and *VA RNA*. Both cells and culture supernatant were harvested at the time points of interest for rAAV8 titration. (B) For the expression of specific AAV proteins using CisReg43CMV-bGpA vector, the gene of interest (GOI) was cloned to the region between human cytomegalovirus (CMV) immediate early enhancer and promoter and beta globin polyA signal (bGpA) region. (C) "+1" indicates that AAP and MAAP were expressed from a 1 base pair frameshift open reading frame in the AAV8 *cap* gene.

**Figure S2. Kinetic profiles of capsids, total intracellular AAV genomes (TG) and VG for VH3B1 and VH3B2 cells under different concentrations of cumate induction.** VH3B1 cells induced with 10 μg/mL doxycycline (10C) and (A) 90 μg/mL cumate (90C), (B) 30 μg/mL cumate (30C) and (C) 15 μg/mL cumate (15C); VH3B2 cells induced with 10C and (D) 90C, (E) 30C and (F) 15C. “VG (C)” denotes the VG titer harvested from cell lysates, while “VG (M)” denotes the VG titer harvested from culture medium. The data are presented as the means±SDs (n=3).

**Figure S3. Distribution of encapsidated rAAV8 produced by rAAV8 triple plasmid transient transfection of HEK293 cells (TriX), VH3, VH3B1 and VH3B2 cell lines.** All the samples were harvested at 72 h post transfection or induction. **“**VG (C)” denotes the VG titer harvested from cell lysates, while “VG (M)” denotes the VG titer harvested from culture medium. “% of Secreted” indicates the percentage of the total VG tier secreted into the culture supernatant. The data are presented as the means±SDs (n=3).

**Figure S4. Expression of adenoviral helper, GFP and ACTB proteins in TriX, VH3, VH3B1 and VH3B2 cell lines.** (A, E): rAAV8 triple plasmid transient transfection of HEK293 cells (TriX); (B, F): VH3 parent cell line; (C, G): VH3B1 cell line; and (D, H): VH3B2 cell line. Human beta-actin proteins encoded by *ACTB* were used as an internal control herein. The data are presented as the means±SDs (n=3).

**Figure S5. Western blot analysis of AAV8 Cap protein in TriX, VH3, VH3B1 and VH3B2 cell lines.** The harvested cells were lysed using NP-40 cell lysis buffer containing protease inhibitors (Roche, Indianapolis, IN, USA). The yielding cell lysates were then clarified using centrifugation and the protein concentration was titrated by bicinchoninic acid (BCA) protein assay (Thermo Fisher Scientific, Waltham, MA, USA). 10 μg of denatured and reduced proteins was loaded to each lane of NuPAGE Bis-Tris gels (Thermo Fisher Scientific, Waltham, MA, USA) and subsequently transferred to the 0.45 μm nitrocellulose membrane (Bio-Rad, Hercules, CA, USA). The blotted membranes were then incubated with mouse anti-AAV VP1/VP2/VP3 antibodies (#65158, PROGEN, Wayne, PA, USA) and anti-GAPDH (Cell Signaling Technology, Danvers, MA, USA) monoclonal antibodies (mAbs) and corresponding anti-mouse IgG conjugated with alkaline phosphatase mAb (Sigma-Aldrich, St. Louis, MO, USA). The chemiluminescent signal was detected using the Immun-StarTM AP Chemiluminescence Kits (Bio-Rad, Hercules, CA, USA). Here, samples were harvested at 48 and 72 h post transfection or induction and followed by total protein extraction.

**Figure S6. Characterization of rAAV2 and rAAV8 producing cell lines.** The copy number of each genetic module, including Genome Module (GM), Replication Module (RM), Packaging Module (PM) and Capsid Module (CM), integrated into the genome of rAAV2-producing GX2/6^1^ and AAV2 VP123-amplified GX6A/B^2^ cell lines as well as rAAV8-producing VH3 and AAV8 VP123-amplified VH3B1/2 cell lines was shown in this figure. In addition, the total intracellular AAV genome titer (TG/cell), vector genome titer (VG/cell), capsid titer (capsid/cell) and full particle content of these producer cell lines under induction by doxcycline (D) at 10 μg/mL and cumate (C) at 10, 30 or 90 μg/mL were listed. Data are presented as the mean values (n=3).

**Figure S7. Overexpression of Rep and adenoviral helper genes enhances AAV8 secretion in VH3B cells.** To construct vectors expressing single AAV Rep or Cap proteins, the coding sequence (CDS) of AAV8 MAAP and AAP, AAV2 Rep78, Rep68, Rep52, and Rep40 were polymerase chain reaction (PCR)-amplified from pAAV2/8 plasmid with an ATG start codon. These DNA segments were then cloned into an in-house custom expression vector called CisReg43CMV-bGpA with CMV promoter and beta globin poly A signal (Figure S1B). All plasmid constructs were verified via DNA sequencing analysis by Integrated DNA Technologies Corp. (Coralville, IA, USA). VH3B2 cells seeded in 6-well plates at a density of 4 × 10^5^ cells per well (7.8 × 10^4^ cells/cm^2^) were transfected with 2.5 μg of pAAV2/8 or pHelper plasmid or in combination with plasmid expressing specific AAV2 Rep or AAV8 Cap protein (map of pAAV2/8 and pHelper plasmids was shown in Figure S1A). After induction for 72 h, both cells and culture supernatant were harvested for VG titer quantification, while the target protein expression was confirmed by proteomics analysis or Western blotting. In the mock control, with addition of polyethylenimine (PEI) but without transient transfection of any plasmid, VH3B2 produced about 6x10^4^ VG/cell but secreted little. Transfection with both pAAV2/8 and pHelper plasmids markedly increased the titer of secreted VG to a level of 60%, similar to that of triple transfection of HEK293 cells. This increase in secretion required the transfection of both pAAV2/8 and pHelper. Either one alone did not boost the secretion of VG to beyond 10%. Transient transfection to boost expression of individual CDS of AAV8 AAP, MAAP and of AAV2 Rep proteins (Rep78, Rep68, Rep52 and Rep40) did not improve secretion either. In addition, boosting both adenoviral helper and individual Rep protein expression together by co-transfection of pHelper plasmid and CDS of Rep78, 68, 52 or 40 slightly increased rAAV8 secretion, but far below the 50% level seen with triple transfection of HEK293 cells. The results suggest that while MAAP is necessary, some unidentified factors are still required for AAV8 secretion. Some of these required factors appear to be present in the pAAV2/8 and pHelper plasmids.

**Table S1. List of heavy isotope-labeled peptides used in the targeted proteomics analysis.**

| Targeted Protein | Heavy isotope-labeled peptide sequence | Reference |
| --- | --- | --- |
| EGFP | FSVSGEGEGDATYG[K_C13N15] | ^1^ |
| Rep78, Rep68 | GIEPTLPNWFAVT[K_C13N15] | ^1^ |
| Rep78, Rep68, Rep52, Rep40 | TAPDYLVGQQPVEDISSN[R_C13N15] | ^1^ |
| DBP | NVSLPVAHSDA[R_C13N15] | ^1^ |
| Beta-actin (encoded by ACTB) | AGFAGDDAPR[R_C13N15] | ^1^ |
| E4orf6 | EELVIL[R_C13N15] | ^2^ |
| AAV8 VP1, VP2, VP3 | NTPVPADPPTTFNQS[K_C13N15] | This study |
| AAV8 VP1 | VLEPLGLVEEGA[K_C13N15] | This study |
| AAV8 AAP | APTEWVVP[R_C13N15] | This study |
| AAV8 MAAP | SGFSNLSVWL[R_C13N15] | This study |

**REFERENCES**

1. Lu M, Lee Z, Lin YC, Irfanullah I, Cai W, Hu WS. Enhancing the production of recombinant adeno-associated virus in synthetic cell lines through systematic characterization. *Biotechnol Bioeng.* 2024;121:341-354.

2. Lu M, Lin YC, Kuo HJ, et al. Tuning capsid formation dynamics in recombinant adeno-associated virus producing synthetic cell lines to enhance full particle productivity. *Biotechnol J.* 2024;19:e2400051.
